# Indium selenide: an insight into electronic band structure and surface excitations

**DOI:** 10.1038/s41598-017-03186-x

**Published:** 2017-06-13

**Authors:** A. Politano, D. Campi, M. Cattelan, I. Ben Amara, S. Jaziri, A. Mazzotti, A. Barinov, B. Gürbulak, S. Duman, S. Agnoli, L. S. Caputi, G. Granozzi, A. Cupolillo

**Affiliations:** 1 Department of Physics, University of Calabria, via ponte Bucci, cubo 31/C, I-87036 Rende, Italy; 2 Theory and Simulation of Materials (THEOS), and National Centre for Computational Design and Discovery of Novel Materials (MARVEL), Ecole Polytechnique Federale de Lausanne, CH-1015 Lausanne, Switzerland; 3 Department of Chemical Sciences, University of Padova, via Marzolo 1, I-35131 Padova, Italy; 4 Laboratoire de Physique de la Matière Condensée, Faculté des Sciences de Tunis, Université de Tunis El Manar, Tunis, 2092 Tunisia; 5 Laboratoire de Physique des Matériaux, Faculté des Sciences de Bizerte, Université de Carthage, 7021 Zarzouna, Tunisia; 6 Elettra-Sincrotrone Trieste S.C.p.A., S.S. 14, km 163.5, I-34149 Trieste, Italy; 7 Department of Physics, Faculty of Sciences, Atatürk University, 25240 Erzurum, Turkey; 8 Department of Basic Sciences, Faculty of Sciences, Erzurum Technical University, 25050 Erzurum, Turkey; 9 School of Chemistry, University of Bristol, Bristol, BS8 1TS United Kingdom; 10 Fondazione Istituto Italiano di Tecnologia, Graphene Labs, Via Morego 30, 16163 Genoa Italy

## Abstract

We have investigated the electronic response of single crystals of indium selenide by means of angle-resolved photoemission spectroscopy, electron energy loss spectroscopy and density functional theory. The loss spectrum of indium selenide shows the direct free exciton at ~1.3 eV and several other peaks, which do not exhibit dispersion with the momentum. The joint analysis of the experimental band structure and the density of states indicates that spectral features in the loss function are strictly related to single-particle transitions. These excitations cannot be considered as fully coherent plasmons and they are damped even in the optical limit, i.e. for small momenta. The comparison of the calculated symmetry-projected density of states with electron energy loss spectra enables the assignment of the spectral features to transitions between specific electronic states. Furthermore, the effects of ambient gases on the band structure and on the loss function have been probed.

## Introduction

Two-dimensional (2D) van der Waals semiconductors^1–4^, combining finite band gaps^5, 6^ and flexibility^7, 8^, are emerging in recent years as the most promising materials for nanoelectronics^9, 10^.

The presence of a band gap, absent in graphene^11^, is crucial for achieving a high ON/OFF ratio in nanodevices^9^. Furthermore, a direct band gap also allows the use of materials in optoelectronics^12^.

Four requisites are crucial for a suitable use of 2D materials in nanotechnology: (*i*) high mobility of charge carriers; (*ii*) the possibility to achieve highly crystalline samples via mechanical/liquid exfoliation; (*iii*) ambient stability; (*iv*) high flexibility together with a sufficiently high fracture toughness. Several classes of materials fail to fulfil the above-mentioned conditions for different motivations: silicene^13^ and germanene^14^ cannot be exfoliated; transition-metal dichalcogenides are characterized by a relatively low value of the mobility of charge carriers^15^; black phosphorus suffers of rapid oxidation in ambient conditions^16^; while bismuth chalcogenides have a poor fracture toughness^17^.

A suitable candidate for nanoelectronics is represented by InSe, which is a layered semiconductor made of stacked layers of Se-In-In-Se atoms with van der Waals bonding between quadruple layers^18, 19^. Recently, many works reported the superb performance of InSe-based optoelectronic devices^20, 21^. Field-effect transistors with an active channel of InSe are characterized by an electron mobility near 10^3^ cm^2^/(V s)^20^ and, moreover, excellent flexibility^22, 23^ and ambient stability^24^, in spite of the presence of a *p*-type doping arising from water decomposition at Se vacancies^24^. Furthermore, InSe is also a promising material for strain engineering^25^, nonlinear optics^26^, and photovoltaics^22^.

However, contrarily to III–V and II–VI semiconductors, indium selenide is not in the main stream of semiconductor literature. To devise broadband photodetectors and, moreover, to assess the suitability of this material for plasmonics, a detailed knowledge of the electronic band structure and of the dielectric response to electromagnetic fields is mandatory.

Depending on the stacking characteristics, three different polytypes (β, ε, γ) of bulk InSe exist^27, 28^. The β (space group symmetry ${D}_{6h}^{4}$) and ɛ (space group symmetry ${D}_{3h}^{1}$) polytypes are characterized by a hexagonal lattice consisting of eight atoms in the unit cell and extending over two layers^29^, whereas rhombohedral γ-polytype (space group symmetry ${C}_{3v}^{5}$) contains two cations and two anions distributed on four adjacent layers^27, 30^.

While ε-InSe has an indirect band gap of 1.4 eV^28^, both β-InSe and γ-InSe have a direct band gap^28^ with nearly identical values of the band gap (1.28^29^ and 1.29^31^ eV, respectively). Thus, only β and γ phases of InSe can be, in principle, used for optoelectronic devices, for which finite and direct band gaps are highly desired^32, 33^.

High-quality samples of β-InSe can be grown by modified Bridgman−Stockbarger method^29^. The possibility to exfoliate β-InSe nanoflakes from a parental bulk single crystal makes this phase suitable for up-scaling, due to the higher ease of the nanofabrication process.

Herein, we report a complete study on the electronic properties of β-InSe by means of angle-resolved photoemission spectroscopy (ARPES), electron energy loss spectroscopy (EELS) and density functional theory (DFT).

EELS offers the possibility to investigate the dielectric response to electron probes enabling non-vertical transitions between occupied and unoccupied states. Thus, EELS studies represent an ideal complement to investigations of the absorption and emission processes of van der Waals semiconductors in the optical limit^34^, i.e., the case of vertical transitions between the valence- and conduction-states.

We observe that damped resonances arising from interband transitions predominate over fully coherent plasmonic excitations in the dielectric response. Moreover, we have identified the interband transitions by comparing the EELS spectra with symmetry-projected density of states (DOS).

## Results and Discussion

### Growth

**Figure 1:**
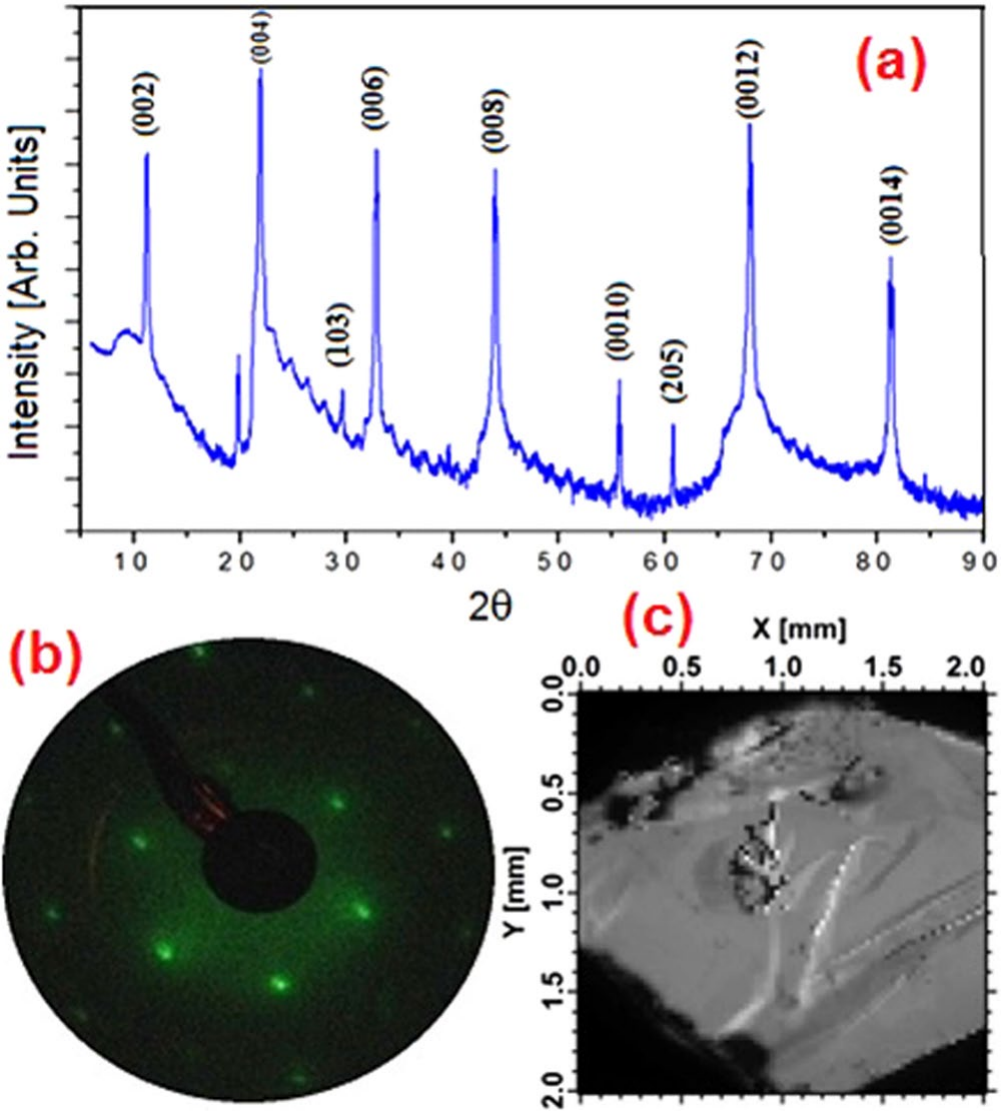
(**a**) XRD spectra of β-InSe. The Miller indices are indicated on each diffraction peak; (**b**) LEED pattern of the as-cleaved sample of β-InSe, acquired at a primary electron energy of 100 eV; (**c**) Topographic map of the photoemission signal of as-cleaved β-InSe at the Γ point of the BZ.

A single-crystal ingot of β-InSe was grown from melt by the modified Bridgman−Stockbarger method as described in the Supplementary Information. The synthesized bulk InSe samples were characterized by X-ray diffraction (XRD, Fig. 1a). Only peaks for hexagonal crystal structure of β-InSe^29, 35^ appear without any other extra peaks, thus indicating the high crystalline purity of the as-grown InSe crystal. From the XRD profiles, the calculated lattice constants are a = b = 4.005 ± 0.004 Å and c = 16.660 ± 0.004 Å, in excellent agreement with previous crystallographic results^36^.

The low-energy electron diffraction (LEED) pattern, shown in Fig. 1b, shows sharps spots, accordingly indicating outstanding surface crystalline quality. The topographic map acquired with photoelectron close to Fermi edge at the Γ point, i.e. normal to the surface plane, (Fig. 1c) shows the presence of large-scale flat terraces, which extend up to hundreds of microns along the surface.

The crystallite size, residual strain and dislocation density have been evaluated by a quantitative analysis of the XRD pattern, as reported in the Supplementary Information.

### Band structure

**Figure 2:**
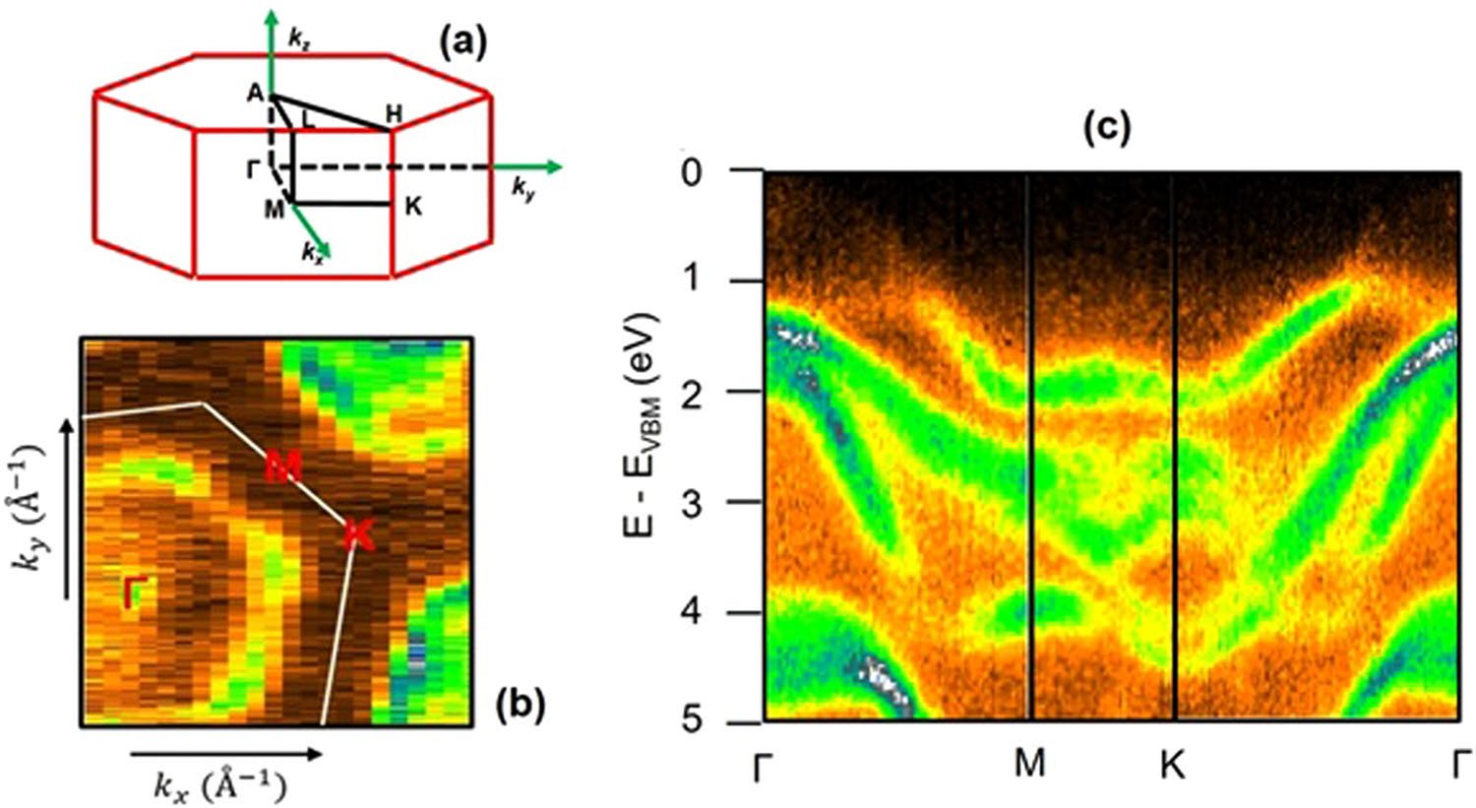
(**a**) Representation of the BZ of β-InSe. (**b**) Constant energy map at 1 eV below the VBM. (**c**) Experimental band structure along the high-symmetry directions. The energy scale was set to zero at VBM.

Figure 2c shows the measured band structure, probed by ARPES, along the high-symmetry directions of the first Brillouin zone (represented in the panels a-b of the same Figure).

At the K point, four major bands with binding energies (BEs) 2.0, 2.6, 3.0 and 4.5 eV are observed, respectively. Along the Γ-M direction, they merge into three bands, whose BE at the M point is 2.0, 2.7, and 3.9 eV.

At the Γ point, two main features are measured at binding energies (BEs) of 1.6 and 4.5 eV, respectively. The latter band crossing Γ at BE 4.5 eV increases its BE along both the Γ-M and Γ-K directions. The band with BE of 1.6 eV at Γ is formed by the merging of three main dispersing bands: (*i*) a first band which decreases its BE at ~1 eV going from Γ to M and then crosses M at 2 eV; (*ii*) a second band which crosses the M point at 2.8 eV with a splitting in the M-K direction into two features which cross K at 2.6 and 3.1 eV; and (*iii*) a third band which increases its BE at 4.1 eV in the Γ-M direction, with a further increase of the BE in the M-K direction up to 4.6 eV. From K to Γ it monotonically increases to 1.6 eV.

**Figure 3:**
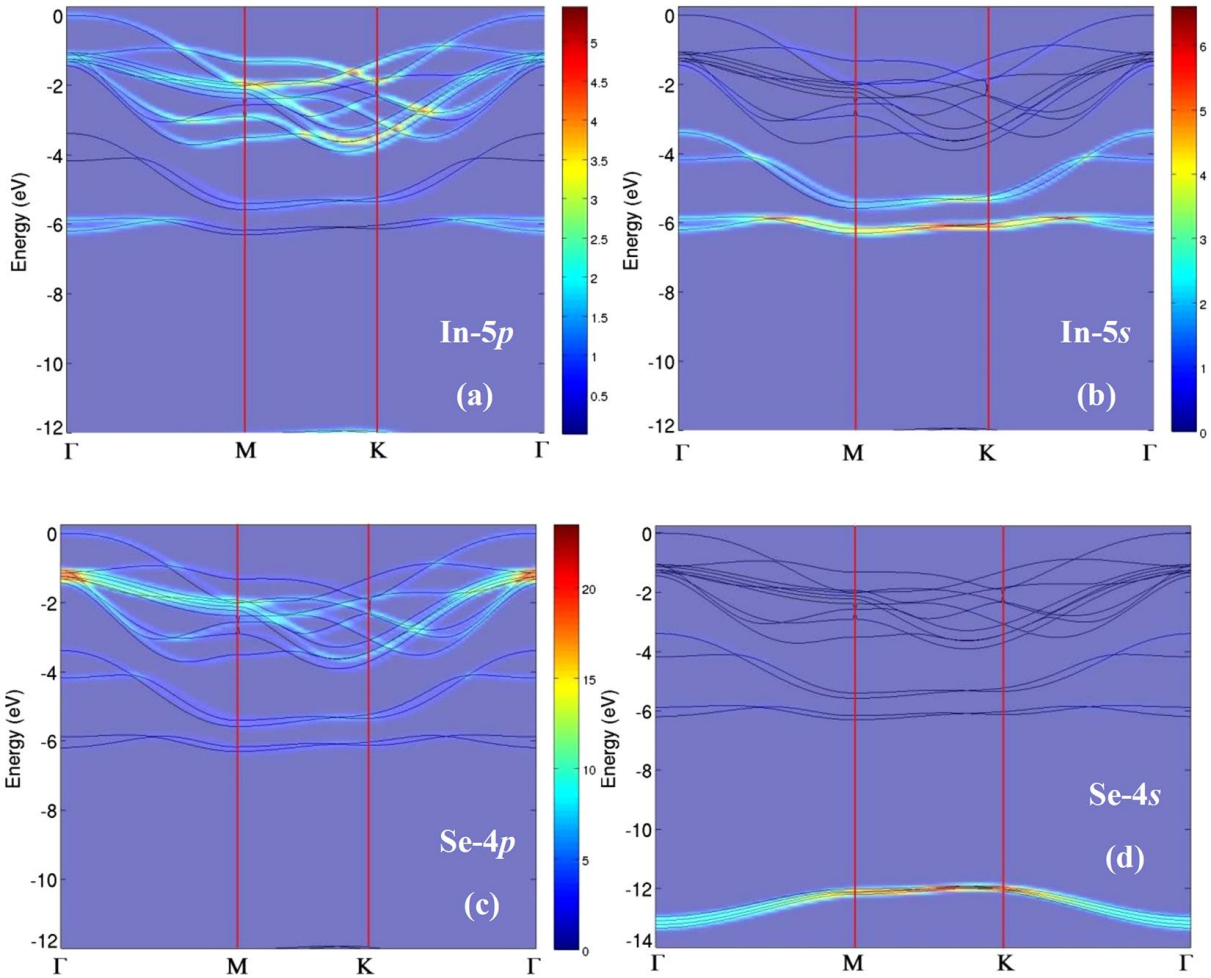
Theoretical band structure projected to (**a**) In-5p; (**b**) In-5s; (**c**) Se-4p; and (**d**) Se-4s atomic orbitals. The intensity of the various bands is plotted in a colour scale, whose legend is reported in the right part of panels (**a**) and (**b**) and between panels (**c**) and (**d**).

The comparison with calculated band structure projected to 5*p* (panel a of Fig. 3) and 5 *s* (panel b) states of In and to 4*p* (panel c) and 4 *s* (panel d) states of Se along the whole BZ, respectively, allows unveiling the main orbital components of the various bands measured by ARPES.

The analysis of experimental (Fig. 2) and theoretical (Fig. 3) band structure indicates that the valence-band maximum (VBM) has a dominant 5*p*_*z*_ component of In. Bands crossing the Γ point at a BE of about 1.6 eV are mainly originated from the Se-4*p* and In-5*s* states, while states at 4.5 eV are principally derived from In-5*s*. Overall, we note that the theoretical model well reproduces the experimental band structure probed by ARPES.

### Loss function probed by EELS and comparison with theory

**Figure 4:**
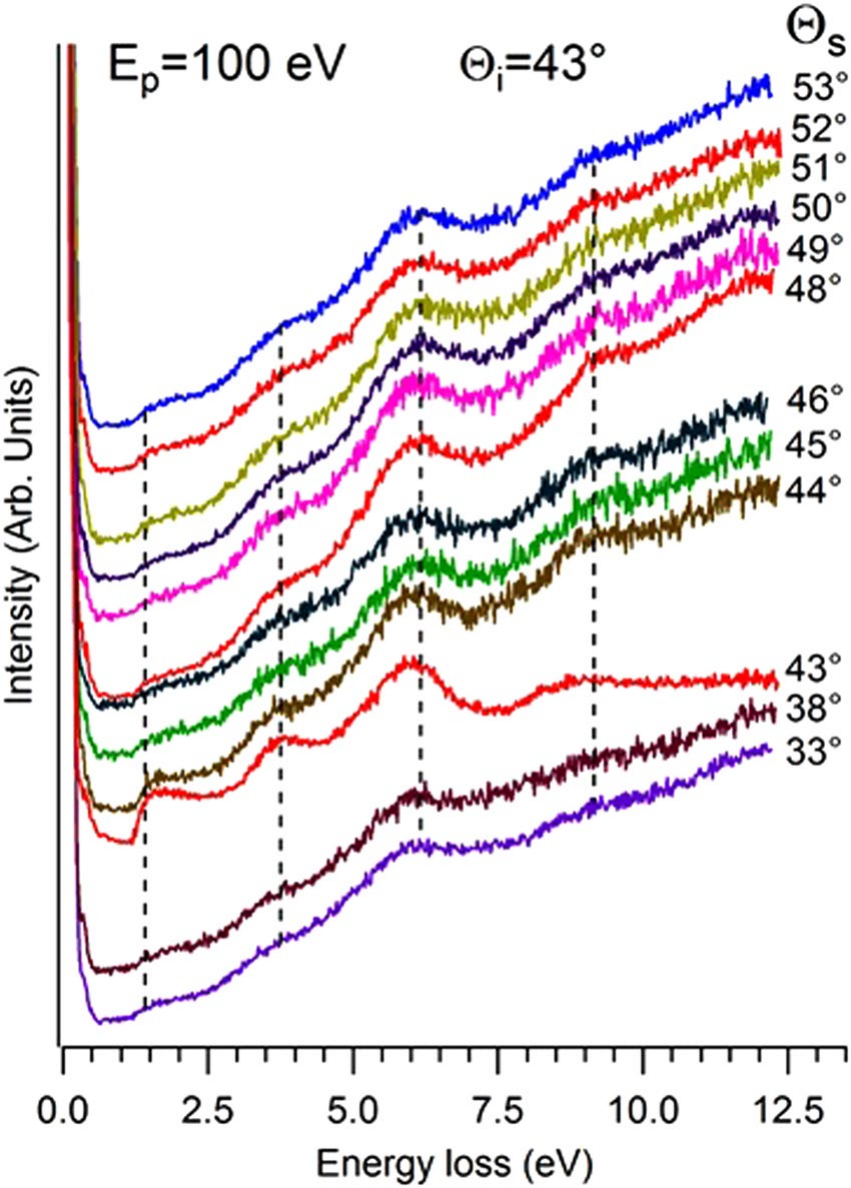
EELS spectra at different values of the scattering angle θ_s_, The corresponding range spanned in the momentum domain is 0–0.79 Å^−1^. The impinging energy is 100 eV.

The angle-resolved EELS spectra of β-InSe sample, measured at different scattering angles with an electron beam energy of 100 eV, are shown in Fig. 4.

A prominent loss feature, located around 6.0 eV, dominates all spectra. Its energy position and intensity is nearly independent on scattering geometry and, consequently, on q_||_ in a momentum range of 0–0.79 Å^−1^ (calculated by means of Equation 2, see Methods). Moreover, weak dispersionless peaks around 1.3, 3.8 and 9.0 eV are revealed.

The experimental loss function probed by EELS can be compared with the calculated loss function *L*(*ω*)^37^, which describes the dielectric response of the system to an incoming electron beam:

1 $$L(\omega )=-Im[\frac{1}{\varepsilon (\omega )}]=\frac{{\varepsilon }_{2(\omega )}}{{\varepsilon }_{1(\omega )+}^{2}{\varepsilon }_{2(\omega )}^{2}}$$

where *ε*_1_(*ω*) and *ε*_2_(*ω*) are the real and imaginary part of the dielectric function *ε*(*ω*) in the framework of the dipole approximation.

**Figure 5:**
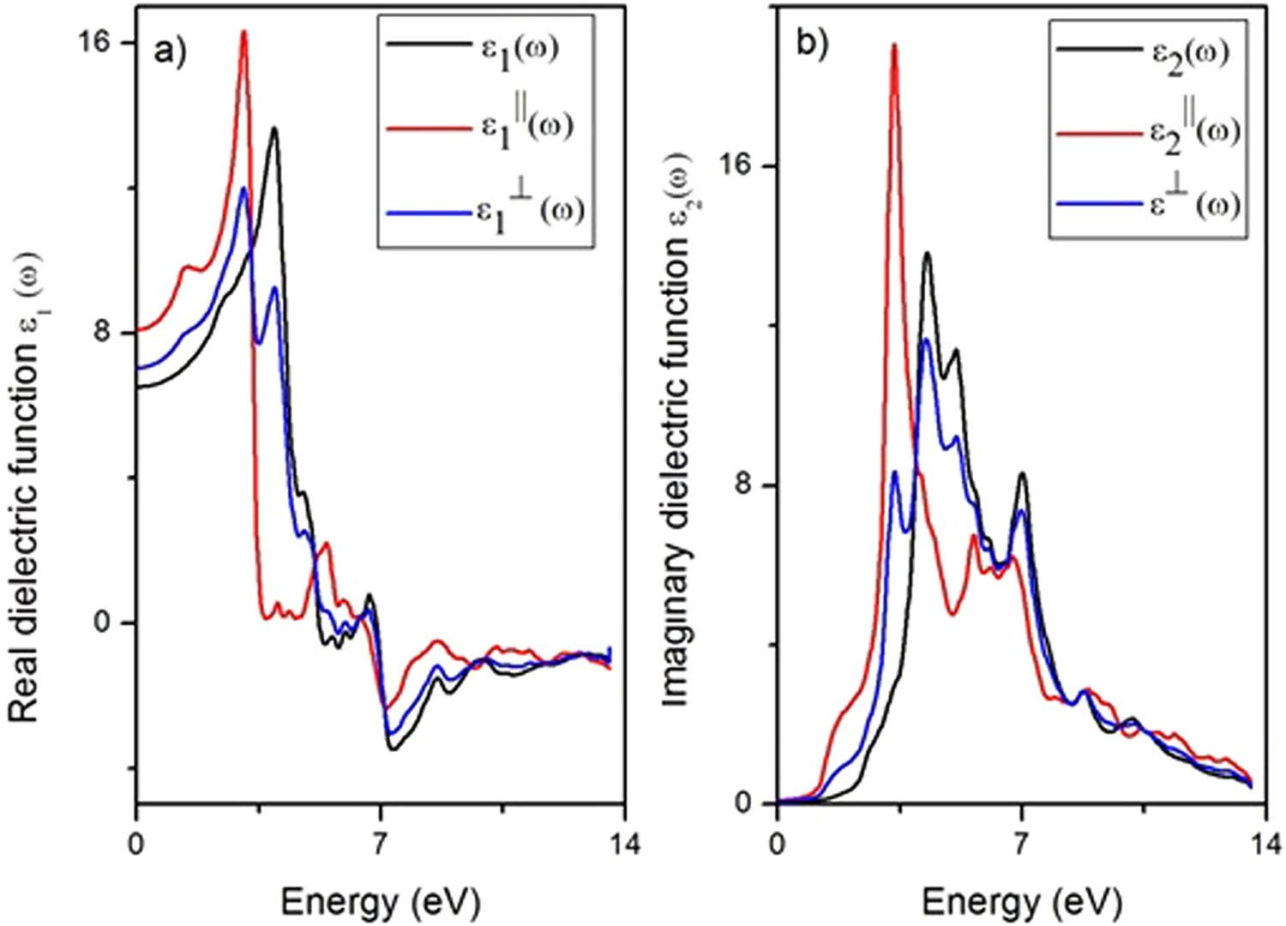
The real (**a**) and imaginary (**b**) parts of the dielectric function obtained from the dielectric theory. Each curve is referred to the total (black), parallel (red) and perpendicular (blue) components, respectively.

In Fig. 5, we report calculations for *ε*_1_(*ω*) (panel a) and *ε*_2_(*ω*) (panel b) decomposed in parallel and perpendicular components, respectively. The parallel and perpendicular part of dielectric function describe the dielectric response of the material to an external electric field with polarization parallel and perpendicular to the surface of the sample, respectively.

The static dielectric constant, i.e. the zero frequency limit of the real dielectric function *ε*_1_(0) (Fig. 5a), which is related to the refractive index *n* measured at low frequencies, is particularly high (${\varepsilon }_{1}^{||}$(0) = 6.4 and ${{\rm{\varepsilon }}}_{1}^{\perp }(\omega )$(0) = 8). In addition, it should be noticed that ${{\rm{\varepsilon }}}_{2}^{\perp }$(ω) and ${{\rm{\varepsilon }}}_{2}^{||}$(ω) in InSe are considerably different. This causes dissimilarities in the dielectric response for different polarizations of the incoming electromagnetic field.

From the analysis of the behavior of the dielectric function of β-InSe with energy (Fig. 5b), we note that the real part of the dielectric function has a finite value in correspondence of the resonances in the EELS spectrum at 3.8 and 9.0 eV, thus they cannot be assigned to plasmonic features. Conversely, the mode at 6.0 eV in principle could be a plasmonic excitation, since the real part of the dielectric function has a pole around 6 eV. However, the imaginary part ε_2_(*ω*) is not small in correspondence of 6 eV, thus hindering the existence of a fully coherent plasmon.

We affirm that features observed in EELS spectrum in Fig. 4 are originated by interband transitions, although the scientific community often refers to these modes as “interband plasmons”^38, 39^, as for the case of the mode associated to π → π* transitions in graphene^40, 41^.

**Figure 6:**
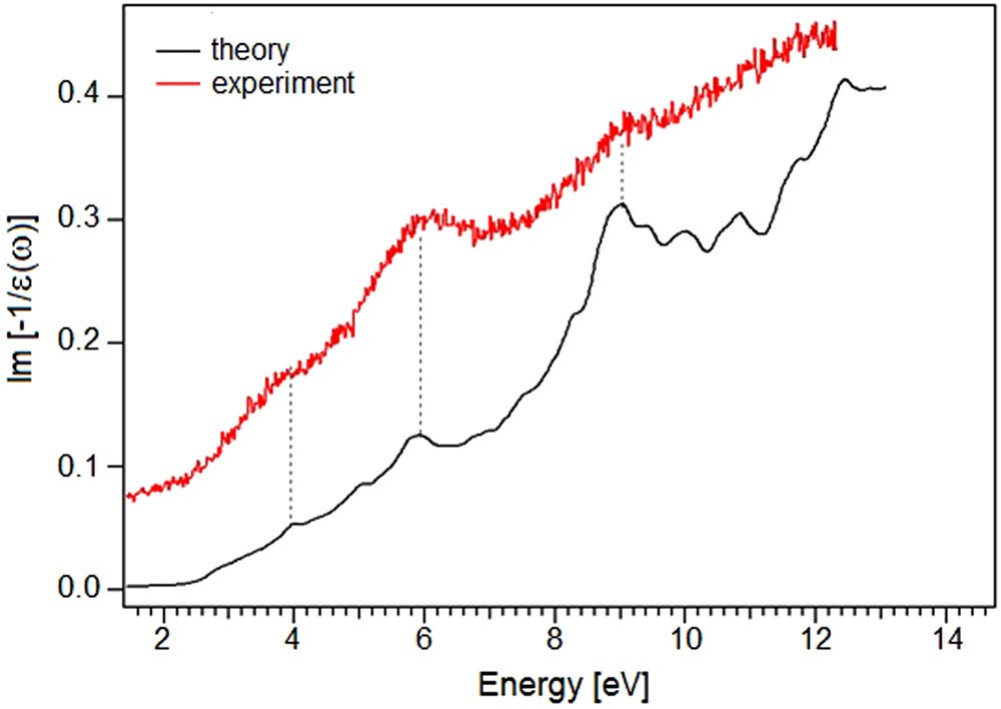
Comparison between the EELS spectrum and the calculated *L* ^||^(*ω*) function.

For the case of reflection EELS (low energy of impinging electrons^42^), the surface contributions are enhanced and, thus, the experimental loss function is well reproduced (Fig. 6) by *L* ^||^(*ω*), i.e. *L*(*ω*) calculated with the parallel components of the dielectric function. Conversely, for EELS in transmission mode (impinging electrons with energy of 100–200 keV^43^) the opposite occurs and *L* ^⊥^(*ω*), i.e. *L*(*ω*) calculated with the perpendicular components of the dielectric function, is more appropriate to describe the electronic response.

The calculated loss function *L* ^||^(*ω*) (black curve in Fig. 6) has null intensity for energy values inferior than the band gap. Several modes are present in the theoretical *L* ^||^(*ω*). In correspondence of the resonances in the excitation spectrum, i.e. energies at which $-Im[\frac{1}{\varepsilon (\omega )}]$ (Fig. 6) shows maxima, the imaginary part of the dielectric function has a nonzero energy value.

To assess the potential exploitation of the resonance at ~6 eV, we have evaluated its quality factor γ(q) = ω(q)/Γ(q)^44^, where Γ(q) is the full-width at half-maximum of the peak. The obtained value, i.e. 4, is just the same estimated for the π-plasmon in graphene (by using data in ref. 45), whose application has been recently proposed for sensors for DNA nucleotides^46^. Thus, one can conclude that the technological potential of the mode at ~6 eV in indium selenide is comparable with that of π-plasmon in graphene.

**Figure 7:**
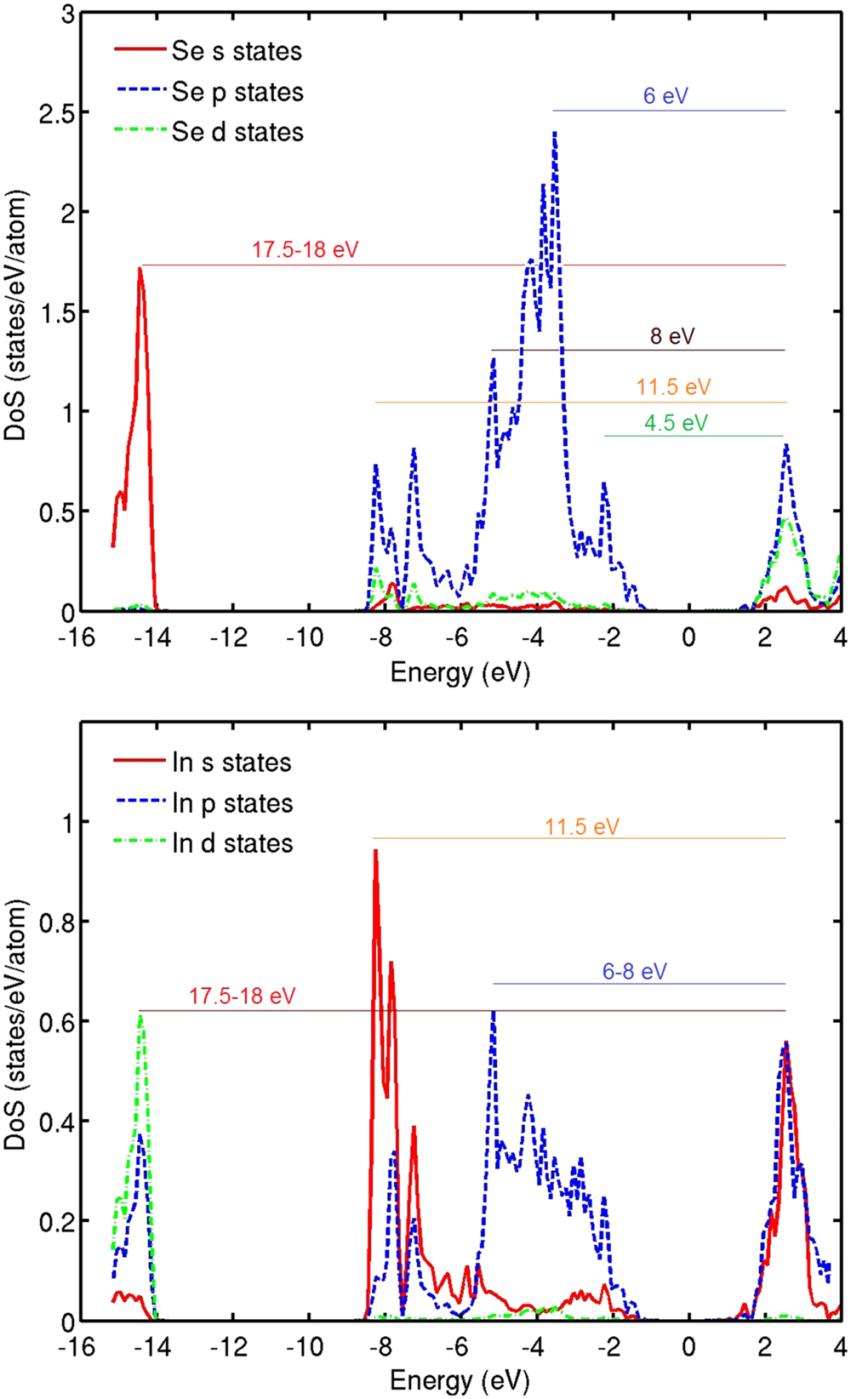
Single-particle transition energies, obtained from our EELS measurements, compared with theoretical DOS (calculated with HSE06) for Se- (top panel) and In-derived (bottom panel) states. Results obtained with the Perdew-Burke-Ernzerhof (PBE) functional are reported in the Supplementary Information.

The comparison of the symmetry-projected DOS and EELS spectra can relate each spectral feature to transitions between specific electronic states (Fig. 7).

The symmetry-projected DOS shows three distinct structures. The energetically lower valence band (VB) is situated between −16 and −14 eV. The upper VB extends from −8 eV up to Fermi level *E*_F_. The third region is that of the conduction band (CB). The state forming the bottom of VB has contributions from *s* states of Se, while the top of the VB has contributions from both *p* states of Se and *sp* states of In, with minor contribution from *p* states of In. The CB has *sp* character, with contributions from both In and Se.

A good agreement between the DOS and modes observed in EELS is found. In details, the peak at a loss energy of ~6 V in EELS spectra should be related to transitions from the 5*p* states of In to unoccupied 6 *s* states of In. The loss peak at ~9 eV measured by EELS is derived from the same transitions, with an additional spectral contribution from transitions from the 4*p* states of Se to unoccupied 5*d* states of Se.

Furthermore, the analysis of the calculated band structure in Figure 3 indicates that the intense bands around 6 and 13 eV below the Fermi energy do not disperse with momentum, in agreement with the dispersionless behavior of modes probed by EELS. The inspection of the DOS suggests the possible occurrence of a mode around 4 eV, related to transitions from 4*p*-derived valence-band states of Se to the 5*d* CB states of Se. The corresponding resonance in the excitation spectrum is observed in our EELS spectra at 3.8 eV (Fig. 4).

We would like to point out that the energies of the EELS peaks cannot be exactly the same of transitions measured with optical methods, since maxima in the loss function correspond to the maxima in $-Im[\frac{1}{\varepsilon (\omega )}]$. On the contrary, optical transitions correspond to the maxima in *ε*_2_(*ω*)^47^, which are shifted compared to maxima of $-Im[\frac{1}{\varepsilon (\omega )}]$ ^37^.

In our EELS spectra in Fig. 4, we also observe another feature around 1.3 eV, ascribed to the formation of direct free excitons in indium selenide^19^. The absence of dispersion of the exciton mode indicates that the momentum dependence of the exchange interaction leads to a flattening of the exciton band structure away from the zone center^48^.

**Figure 8:**
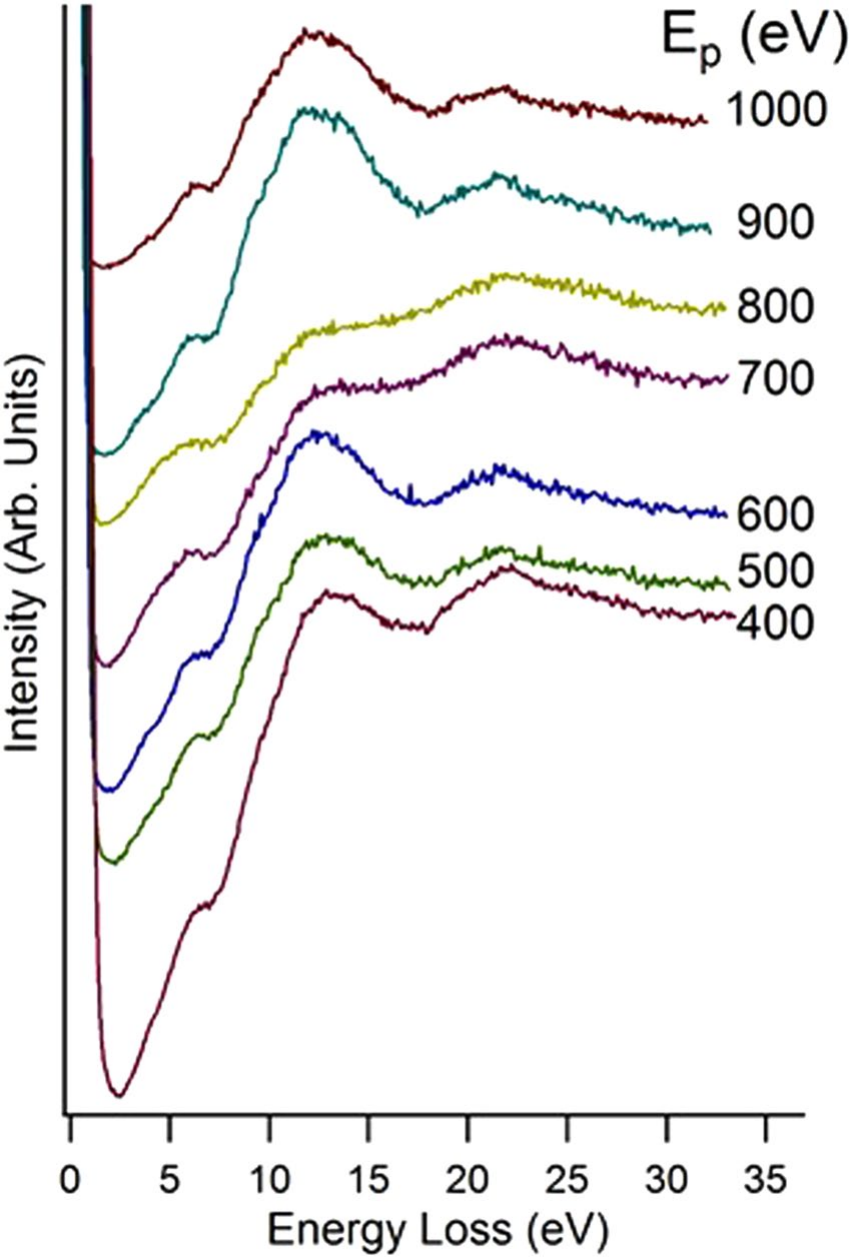
EELS spectra acquired at different values of the primary energy E_p_.

By increasing the impinging energy of the electron beam, it is possible to extend the excitation spectrum, including also modes at higher loss energies. Figure 8 reports the EELS spectra acquired at different values of *E*_*p*_, ranging from 400 to 1000 eV, in an extended energy loss range (0–34 eV) with respect to EELS measurements at *E*_*p*_ = 100 eV in Fig. 4. We observe two additional peaks at 12 and 22 eV, while the mode at 8 eV has decreased weight, likely due to the modification of the cross-section for its excitation at higher values of *E*_*p*_. The mode at 12 eV has the highest intensity in the EELS spectrum acquired with *E*_*p*_ in the 400–1000 eV range. By examining the theoretical DOS, we ascribe it to single-particle transitions with contribution from 5 *s* → 5*p* between In-derived states and from 4*p* → 5*d* between Se-derived states. Moreover, the interplay of different excitations in the 11.5–14.0 eV range explains the broadness of the experimental peak, as well as its high intensity.

### Surface chemical reactivity and ambient stability

**Figure 9:**
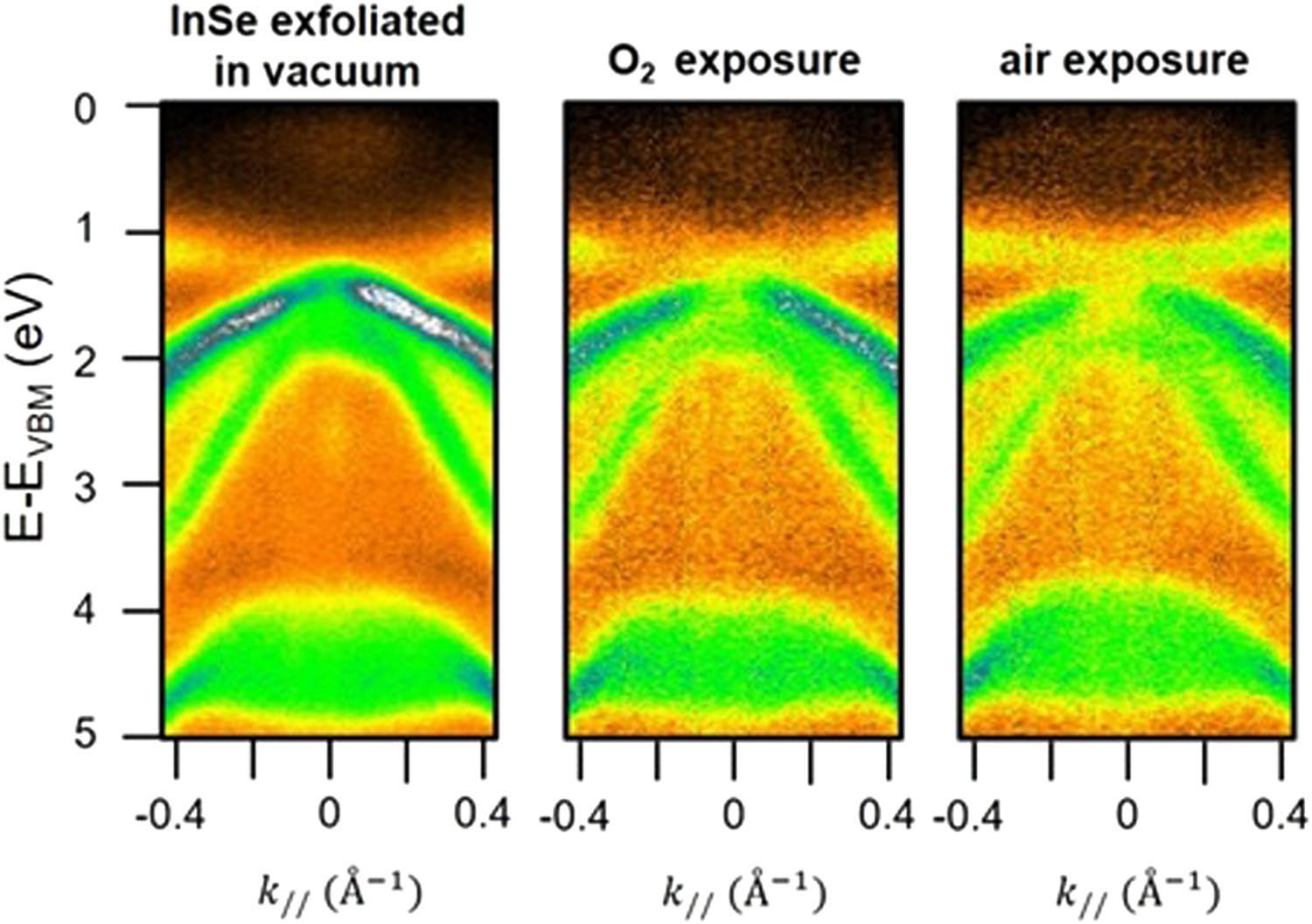
InSe band structure close to the Г point for the as-exfoliated sample in UHV (left panel) and its modification upon O_2_ (~10^3^ L, central panel) and air exposure (right panel) at room temperature. The energy scale was set to zero the VBM of the as-exfoliated sample.

Ambient stability would be an essential prerequisite for up-scaling of InSe-based devices. In order to study the robustness of the InSe band structure, the sample, exfoliated in ultra-high vacuum (UHV) conditions, was exposed to ~10^3^ L (1 L = 10^−6^ torr ∙ s) of O_2_ and to air at room temperature. The band structure (Fig. 9) does not show any remarkable change, thus pointing to a good ambient stability of InSe. In details, exposure to O_2_ under UHV conditions does not induce any shift of the electronic bands of pristine InSe and, moreover, no oxygen-induced bands emerge. Remarkably, only an overall slight decrease of the photoemission signal has been observed in the air-exposed sample, due to the presence of chemisorbed species coming from water decomposition^24^, whose presence attenuates the signal produced by outgoing photoelectrons. By contrast, rapid surface degradation in ambient conditions has been reported for the cases of black phosphorus^16^, Bi_2_Se_3_^49^, and MoS_2_^50^.

Upon air exposure, a *p*-doping of 130 meV is estimated by inspecting the rigid shift of the bands in Fig. 9 towards lower BE. The *p*-type doping of the air-exposed InSe well agrees with the reported *p*-type behaviour of uncapped InSe nanodevices, due to water decomposition at Se vacancies^24^. The interaction with ambient humidity does not introduce other modifications in the band structure, contrarily to the case of Bi_2_Se_3_^51^.

Formerly, *p*-type doping has been reported for graphene exposed to ambient air humidity^52^. As regards transition-metal dichalcogenides, H_2_O-induced *n*-type depletion has been observed in MoS_2_ and MoSe_2_^6^, whereas the reverse takes place in WSe_2_^6^.

**Figure 10:**
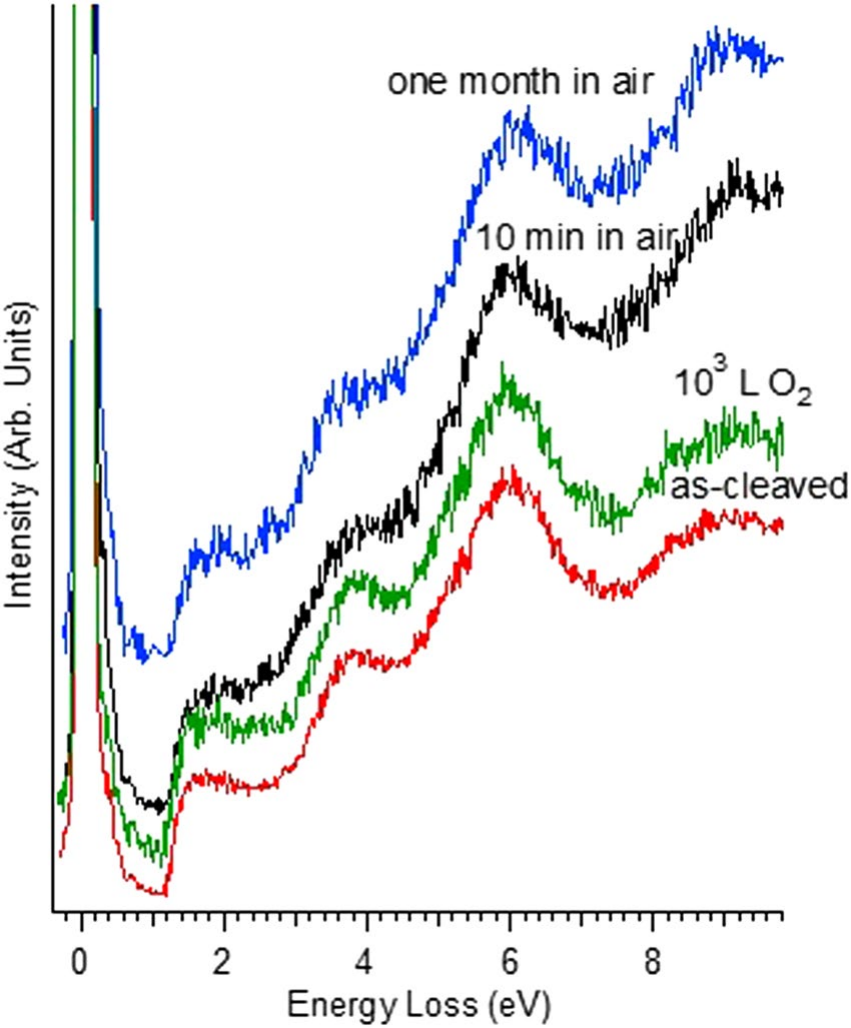
Evolution of the loss function of the as-cleaved InSe sample (red curve) upon exposure to O_2_ (10^3^ L, green curve) and after 10 minutes (black curve) and one month (blue curve) in air. All EELS experiments and exposures have been made at room temperature. The impinging energy is 100 eV. The incidence and the scattering angles are 43° with respect to the sample normal.

In Fig. 10, the evolution of the loss function upon O_2_ and air exposure is shown. No perceptible change can be appreciated by EELS, even after one month in air, except a slight modification of the background.

On the basis of ARPES and EELS data in Figs 9 and 10, we can affirm that the β-InSe sample is stable in atmospheric conditions and scarcely reactive to ambient gases, due to the protection of the Se termination of the crystal.

## Conclusions

We have studied the electronic properties of indium selenide by means of a combination of spectroscopic tools and DFT. The orbital character of the various bands, measured by ARPES, has been established by means of a comparison with the symmetry-projected band structure, calculated by DFT. The loss function, probed by EELS, shows the direct free exciton and several dispersionless features, all ascribed to resonances originated from interband transitions, which predominate over plasmonic modes. In addition, we find that exposure to ambient atmosphere introduces a *p*-type doping by 130 meV, i.e. a rigid shift in the VB. However, the band structure is stable in ambient conditions without the emergence of oxygen- or water-induced adsorbate bands. Ambient stability is crucial in the prospect of InSe-based nanoelectronics and optoelectronics.

## Methods section

### Experimental methods

The electronic structure in the VB was investigated by ARPES at the Spectromicroscopy beamline at Elettra synchrotron (Trieste, Italy). ARPES data were acquired with a photon energy of 74 eV using a hemispherical electron energy analyser with angular resolution of 0.33°. The sample was mounted onto a scanning stage, which enables positioning and raster imaging with respect to the fixed photon beam. Photoelectron intensity distribution maps I *(k*_*x*_ *, k*_*y*_, E) were taken from microscopic areas (the beam size was about 1 µm) by rotating the electron energy analyser with respect to the sample using a two-axis goniometer. The ARPES measurements were acquired at about 110 K with an energy resolution of ≈50 meV.

The angle-resolved EELS measurements were carried out at room temperature using an EELS apparatus with two 50 mm hemispherical deflectors for both monochromator and rotating analysers, mounted in an UHV chamber at University of Calabria, Italy. The incident electron beam impinges on the sample at a fixed angle of incidence *θ*_*i*_ = 43° with respect to the surface normal, along the ГK direction of the surface BZ. The primary electron beam energy *E*_*p*_ is 100 eV. The parallel momentum transfer ${q}_{||}$was calculated from the following kinematic expression, which reflects the conservation of energy and of in-plane momentum^53^:

2 $${q}_{||}=[\sin \,{\theta }_{i}-{(1-\frac{{E}_{l}}{{E}_{p}})}^{\frac{1}{2}}\,\sin \,{\theta }_{s}]{(\frac{2m{E}_{p}}{{\hslash }^{2}})}^{\frac{1}{2}}$$

where *E*_l_ is the loss energy, *m* is the free-electron mass and *θ*_s_ is the scattering angle with respect to the surface normal of the sample.

EELS experiments as a function of the primary electron beam energy *E*_p_ were acquired in specular geometry in a broad energy range (100–1000 eV).

### Theoretical Methods

The electronic ground state properties of β-InSe were calculated using DFT, as implemented in the QUANTUM-ESPRESSO package^54^, using ultrasoft pseudopotentials^55^ with a 4*s* ^2^4*p* ^2^ valence configuration for Se and including semicore *d* states (4*d* ^10^5*s* ^2^5*p* ^1^) for In. Both the generalized gradient PBE^56^ approximation and the Heyd-Scuseria-Ernzerhof (HSE06)^57, 58^ hybrid functionals were used for the exchange-correlation term. The electronic wave functions were expanded in plane waves up to a 60 Ry energy cutoff and a 600 Ry charge density cutoff. The integration over the BZ was performed using a 12 × 12 × 4 Monkhorst-Pack mesh^59^. We fixed the cell geometry at the experimental lattice parameters found by XRD (see Fig. 1a and its related discussion) and the atomic positions were relaxed until the forces were below a 5·10^−5^ a.u. threshold. A van der Waals correction according to the DFT-D scheme^60^ was added in the case of the PBE functional.

To calculate the dielectric response, based on self-consistent scheme, we have applied the all electron Full-Potential Linearized Augmented Wave plus local orbital (FP-LAPW + lo)^61^, as embedded in WIEN2k simulation package^62^, to solve the Kohn–Sham equations^63^. The dielectric function *ε*(*ω*) = *ε*_1_(*ω*) + i*ε*_2_(*ω*) has been calculated by using the generalized gradient approximation functional developed by Engel and Vesko (EV)^64^.

The β-InSe compound, having hexagonal symmetry, has two non-zero diagonal components of the dielectric tensor. These components correspond to an electric field perpendicular and parallel to the z-axis, namely, ${{\rm{\varepsilon }}}_{1}^{xx}({{\rm{\varepsilon }}}_{1}^{\perp })$ and $\,{{\rm{\varepsilon }}}_{1}^{zz}({{\rm{\varepsilon }}}_{1}^{||})$.

The imaginary dielectric function *ε*_2_(*ω*) is computed by summing all possible direct interband transitions between the occupied and empty states, taking into account the appropriate momentum matrix elements, by means of the following expression (see also Supplementary Information):

3 $${{\rm{\varepsilon }}}_{2}^{ij}({\rm{\omega }})=\frac{8{{\Pi }}^{2}{e}^{2}}{{\rm{\Omega }}{m}^{2}{{\rm{\omega }}}^{2}}\sum _{c}^{unocc}\sum _{v}^{occ}{\int }_{BZ}{|{M}_{cv}(k)|}^{2}{f}_{vk}(1-{f}_{ck}){\rm{\delta }}\,({E}_{ck}-{E}_{vk}-{\hbar}{\rm{\omega }})\,{d}^{3}k$$

The momentum matrix elements *M*_*cv*_(*k*) are the components of the momentum operator p (p = −iħ∇): ${M}_{cv}(k)=\langle ck|\nabla .{\rm{\delta }})\,|vk\rangle$, where δ is the potential vector defining the applied electric field. They are calculated from the occupied (valence) and the unoccupied (conduction) electron states (*ck*,*vk*) and the integration over the irreducible BZ is performed to calculate *ε*_2_ (ω). The Fermi distribution function *f*_*vk*_ is used to evaluate the transitions from occupied to unoccupied states. The term δ(*E*_*ck*_ − *E*_*vk*_ − *ħω*) indicates the conservation condition of total energy. The integration over the BZ is improved by the tetrahedron method^65^.

Furthermore, we have used the Kramer-Kronig transformation to calculate the real dielectric function^66^:

4 $${ {\mathcal E} }_{1}(\omega )=1+\frac{2}{\pi }{\rm{P}}{\int }_{0}^{\infty }\frac{{\rm{\omega }}^{\prime} { {\mathcal E} }_{2}({\rm{\omega }}^{\prime} )}{{{\rm{\omega }}^{\prime} }^{2}-{{\rm{\omega }}}^{2}}{\rm{d}}{\rm{\omega }}^{\prime} $$

where P is the principle value of the integral.

## Electronic supplementary material

Supplementary Information
