# Supplementary material for: Indium selenide: an insight into electronic band structure and surface excitations
Source: Sci Rep. 2017 Jun 13;7:3445. doi: 10.1038/s41598-017-03186-x (PMC5469805; doi:10.1038/s41598-017-03186-x)
Supplement: Supplementary file 1 — Supplementary Information [file 41598_2017_3186_MOESM1_ESM.pdf]

Supplementary Information

Indium selenide: an insight on electronic band structure and surface excitations

A. Politano, D. Campi, M. Cattelan, I. Ben Amara, S. Jaziri, A. Mazzotti, A. Barinov, B. Gürbulak, S. Duman, S. Agnoli, L. S. Caputi, G. Granozzi, and A. Cupolillo

Growth of single-crystal InSe

InSe crystals were grown by using the Bridgman-Stockbarger method. The melting point of 660±5 °C of the InSe compound was determined from the phase diagram.

In and Se starting elements of a high purity grade (not less than 99.999%) were sealed in quartz ampoule, which was annealed at 950 °C under a vacuum of 10^{-6} mbar for 15 hours in a furnace. The temperature of the quartz ampoule was decreased to room temperature in 24 hours. The crucible was then suspended in the middle of the vertical furnace with two zones designed. The temperature of the furnace was increased to 950 °C and maintained at this temperature for 40 hours and then decreased to 750 °C. Temperature was kept at 750°C for 15 hours. The temperature of the lower zone of furnace was reduced to 250 °C at a rate of 1.54 °C/h. Both the furnace zones were cooled to 250 °C in 75 hours. The solidified ingot was cooled to room temperature in 50 hours. The grown InSe samples are 10 mm in diameter and about 60 mm in length. Samples were cleaved into perpendicular planes parallel to the (001) surface with a razor.

X-ray diffraction (XRD)

The experimental interplanar distance (d_{exp}) values has been calculated by using Bragg diffraction law. The values of standard interplanar distance (d_0), d_{exp} and intensity ratio data are given in Table S1

Table S1. The standard and calculated XRD results for the undoped InSe

Peak (hkl)	2θ	Intensity (a.u.)	d_0 (Å)	d_{exp}.(Å)
(002)	11.19	72009	7.910	7.900
(004)	21.89	310787	4.068	4.060
(103)	29.71	827	3.010	3.004
(006)	32.90	77577	2.720	2.710
(008)	44.09	53923	2.055	2.052
(0010)	55.75	1008	1.650	1.640
(0010)	60.84	427	1.523	1.521
(205)	68.05	132001	1.379	1.376
(0012)	81.32	10236	1.184	1.182

The peak width at half maximum used to determine the crystallite size (*D*) by using Debye–Scherrer formula is:

$$D = \frac{K\lambda}{(\beta cos\,\theta\,)} \qquad (2)$$

where *K*=0.94 is the Scherrer constant, β is the full-width at half maximum (FWHM) of InSe diffraction peak.

The strain (ε) and dislocation density (δ) values for InSe are calculated for all peaks by Eq. (3) and Eq. (4) [1]:

$$\varepsilon = \frac{(\beta cos\theta)}{4} \qquad (3)$$

$$\delta = \frac{15\varepsilon}{(axD)} \qquad (4)$$

The crystallite size, residual strain and dislocation density and values for the InSe semiconductor calculated using the Eq. (2), Eq. (3) and Eq. (4) are presented in Table S2.

Table S2. The FWHM, crystallite size (D), dislocation density (ε) and residual strain (δ) values for the InSe samples.

**Peak (hkl)**	**2θ**	**FWHM**	**$D_{exp.}$ (Å)**	***ε*, ($lin^{-2}m^{-4}$) x10^{-4}**	***δ*, (lin/m^{-2}) x10^{14}**
(002)	11.19	0.156	534.8	6.7	2.86
(004)	21.89	0.238	355.3	10.19	7.92
(103)	29.71	0.074	1126.3	3.2	1.26
(006)	32.90	0.097	885.5	4.09	1.27
(008)	44.09	0.153	585.9	6.18	2.91
(0010)	55.75	0.072	1304.2	2.77	0.587
(205)	60.84	0.098	983.2	3.68	1.03
(0012)	68.05	0.060	1670.9	2.16	0.356
(0014)	81.321	0.121	905.0	4.0	1.22

DOS calculation with the PBE

Figure S1 reports the theoretical DOS (calculated with the PBE functional) for In- and Se-derived states.

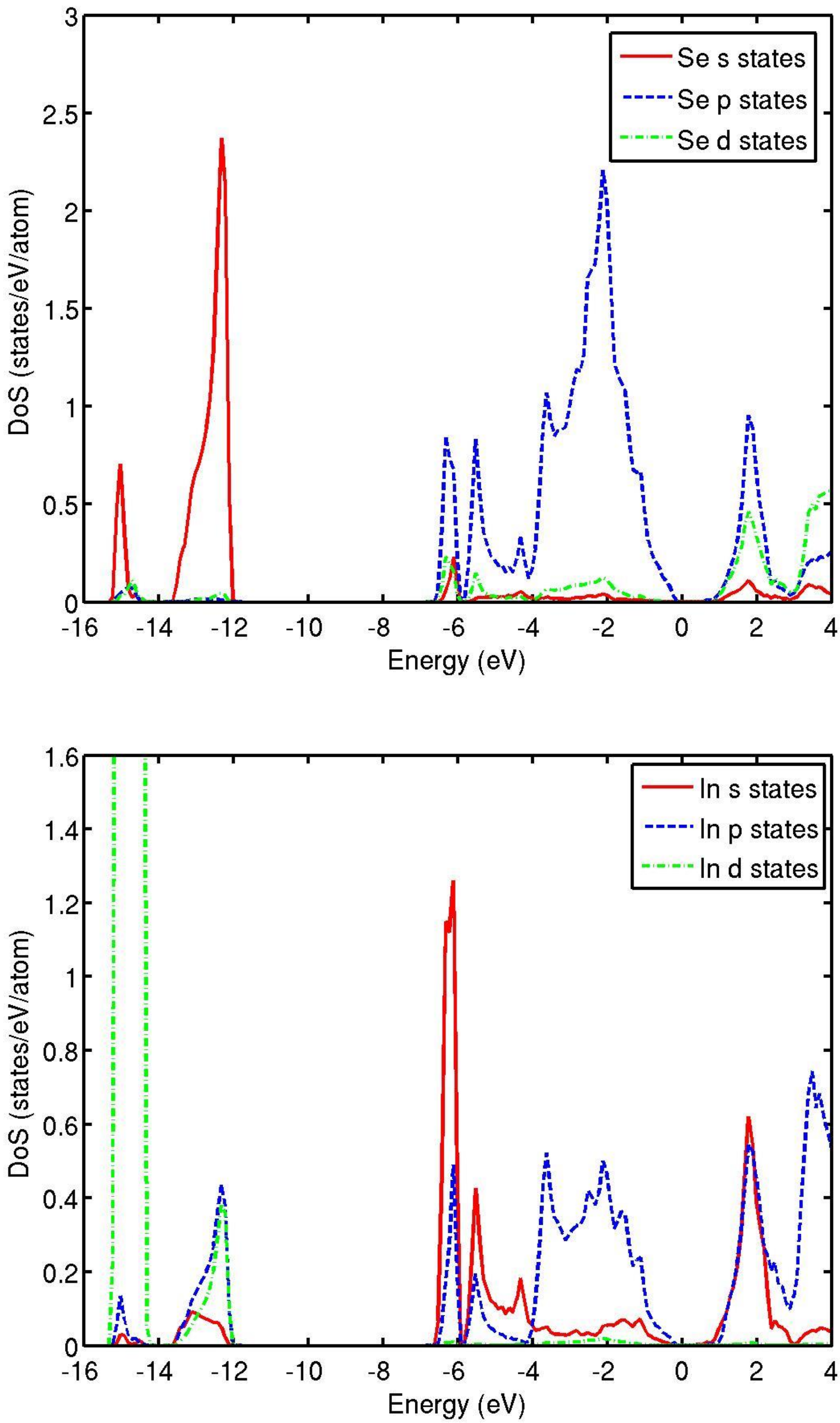

Figure S1: Theoretical DOS (calculated with PBE) for Se- (top panel) and In-derived (bottom panel) states.

Dielectric theory

In the limit of linear response, the interaction between the external perturbing electric field and the material is fully described by the complex dielectric function $\varepsilon(\omega, q)$.

This function contains all the information necessary to compute the electron energy-loss spectra as described in Ref. 2 In this paper, we compute the imaginary part of the dielectric function using Equation (3) which derives from the general definition of the dielectric response of a material in the frame of random phase approximation (RPA) using the expression of the independent particle polarizability given by Hedin.[3] and assuming the so called optical approximation limit of $q \rightarrow 0$. The applicability of such $q \rightarrow 0$ limit in our case relies on the fact that the employment of fast electrons in the impinging beams enables only the transfer of small momenta q in the inelastic interaction process. The real part of the dielectric function is then computed via Kramers-Kronig relations. Detailed descriptions of the derivation and the physical meaning of Equation (3) can be found in Ref. 4. Specific aspects of its implementation in the Wien2K code has been reported by Ambrosh-Draxl and Sofo [5]. Examples of the application of Equation (3) to the calculation of EELS spectra can be found in Ref. 6.

Equation (3) of the main text has been obtained starting from Ref. 7 by considering the spin degeneracy.

Finally, we note that within the dipole approximation, $\varepsilon_2(\omega)$ is given in the momentum representation in terms of indirect and direct interband transitions, where the transitions satisfying the dipole selection rule $\Delta l = \pm 1$ are considered. However, the indirect ones involve phonon scattering, which is neglected due to its small contribution to $\varepsilon_2(\omega)$.

REFERENCES

1 Abdullah, M. M., Bhagavannarayana, G. & Wahab, M. A. Growth and characterization of GaSe single crystal. *J. Cryst. Growth* **312**, 1534-1537 (2010).

2 Rivacoba, A., Zabala, N. & Aizpurua, J. Image potential in scanning transmission electron microscopy. *Prog. Surf. Sci.* **65**, 1-64 (2000).

3 Hedin, L. New method for calculating the one-particle Green's function with application to the electron-gas problem. *Phys. Rev.* **139**, A796 (1965).

4 Wooten, F. *Optical Properties of Solids*. (1972).

5 Ambrosch-Draxl, C. & Sofo, J. O. Linear optical properties of solids within the full-potential linearized augmented planewave method. *Comput. Phys. Commun.* **175**, 1-14 (2006).

6 Mauchamp, V., Boucher, F., Ouvrard, G. & Moreau, P. Ab initio simulation of the electron energy-loss near-edge structures at the Li K edge in Li, Li_2O, and $LiMn_2O_4$. *Phys. Rev. B* **74**, 115106 (2006).

7 Reshak, A. *et al.* Structural, electronic and optical properties in earth-abundant photovoltaic absorber of Cu_2ZnSnS_4 and $Cu_2ZnSnSe_4$ from DFT calculations. *Int. J. Electrochem. Sci.* **9**, 955-974 (2014).
